# Radiation Dose Reduction Efficiency of Buildings after the Accident at the Fukushima Daiichi Nuclear Power Station

**DOI:** 10.1371/journal.pone.0101650

**Published:** 2014-07-07

**Authors:** Satoru Monzen, Masahiro Hosoda, Minoru Osanai, Shinji Tokonami

**Affiliations:** 1 Department of Radiological Life Sciences, Division of Medical Life Sciences, Graduate School of Health Sciences, Hirosaki University, Hirosaki, Aomori, Japan; 2 Research Institute for Radiation Emergency Medicine, Hirosaki University, Hirosaki, Aomori, Japan; Enea, Italy

## Abstract

Numerous radionuclides were released from the Fukushima Daiichi Nuclear Power Station (F1-NPS) in Japan following the magnitude 9.0 earthquake and tsunami on March 11, 2011. Local residents have been eager to calculate their individual radiation exposure. Thus, absorbed dose rates in the indoor and outdoor air at evacuation sites in the Fukushima Prefecture were measured using a gamma-ray measuring devices, and individual radiation exposure was calculated by assessing the radiation dose reduction efficiency (defined as the ratio of absorbed dose rate in the indoor air to the absorbed dose rate in the outdoor air) of wood, aluminum, and reinforced concrete buildings. Between March 2011 and July 2011, dose reduction efficiencies of wood, aluminum, and reinforced concrete buildings were 0.55±0.04, 0.15±0.02, and 0.19±0.04, respectively. The reduction efficiency of wood structures was 1.4 times higher than that reported by the International Atomic Energy Agency. The efficiency of reinforced concrete was similar to previously reported values, whereas that of aluminum structures has not been previously reported. Dose reduction efficiency increased in proportion to the distance from F1-NPS at 8 of the 18 evacuation sites. Time variations did not reflect dose reduction efficiencies at evacuation sites although absorbed dose rates in the outdoor air decreased. These data suggest that dose reduction efficiency depends on structure types, levels of contamination, and evacuee behaviors at evacuation sites.

## Introduction

Numerous radionuclides were released from the Fukushima Daiichi Nuclear Power Station (F1-NPS) following the magnitude 9.0 earthquake and tsunami on March 11, 2011. The most contaminated area in the Fukushima Prefecture was the northwest of F1-NPS [1], [2]. In total, approximately 154,000 people were evacuated from Fukushima, of which 109,000 were within the “Evacuation Order Area.” Approximately 86,000 of these people have been evacuated as of February 2014 [3], and local residents are eager to calculate their individual radiation exposure.

To support radiation surveying and contamination monitoring of safe shelters immediately after the first hydrogen explosion, Hirosaki University sent many staff members to the temporary safe shelters that were set up around F1-NPS [4]. Almost all of these shelters were public facilities such as schools and exhibition halls, and they had various structure types (e.g., wood, aluminum, or reinforced concrete construction). Absorbed dose rates in the indoor and outdoor air at 18 of these sites were measured using a gamma-ray measuring devices, and individual radiation exposure was calculated by assessing the radiation reduction efficiency of each structure type. Absorbed dose rates in the indoor and outdoor air were monitored at each evacuation site in Fukushima for 5 months after the accident, and data were collected to assess the impacts of time, direction, and distance from F1-NPS on the dose reduction efficiency of the structure types at each of these sites.

## Materials and Methods

### Planning for measured location of evacuation sites

**Figure 1:**
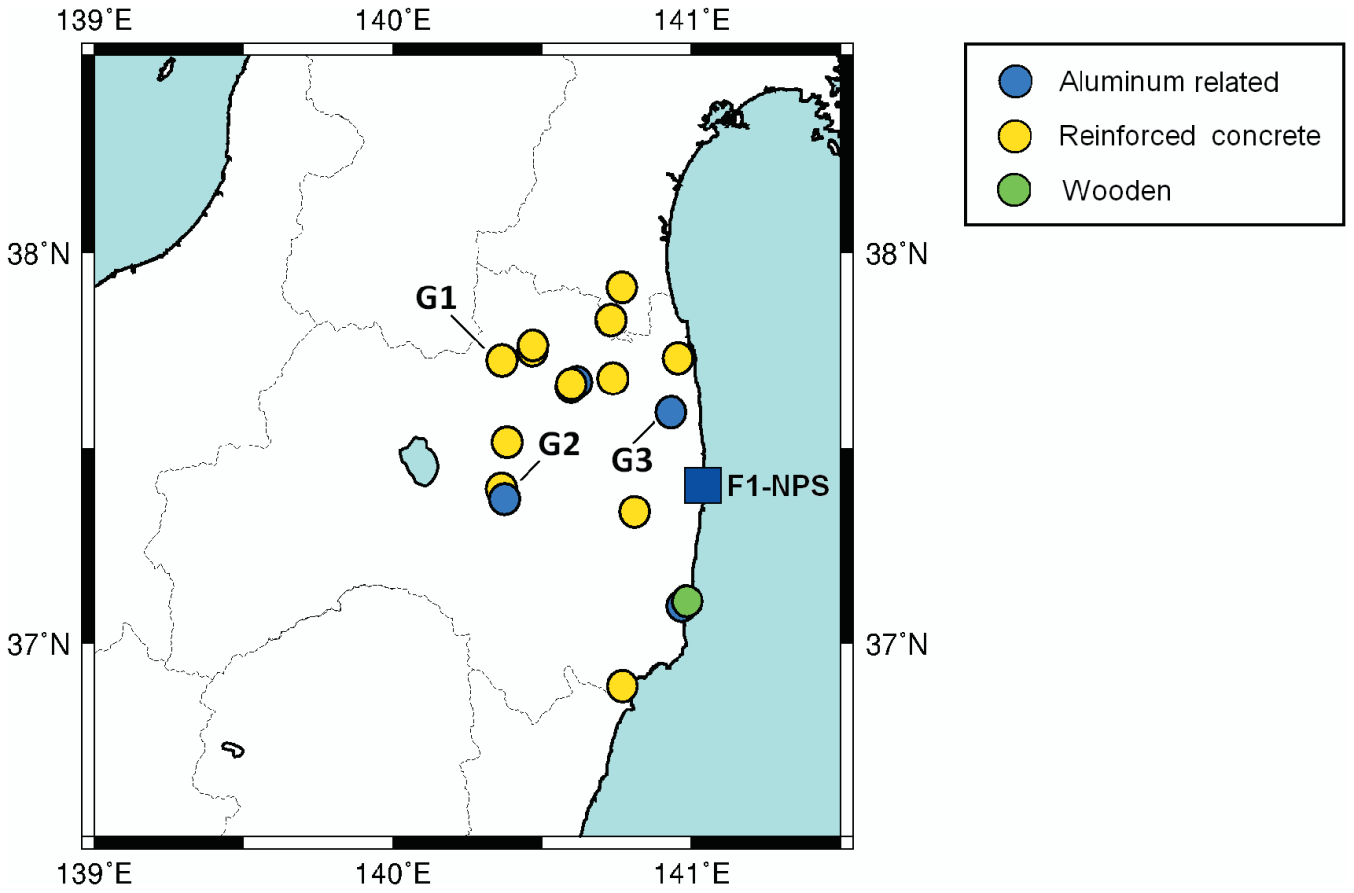
Evacuation sites examined for absorbed dose rates in the indoor and outdoor air in the Fukushima Prefecture. Evacuation sites were 21–64 km from F1-NPS, and the buildings were aluminum-related (blue), reinforced concrete (yellow), and wooden (green) constructions. The map was generated using GPS data from each site. Markers of G1–G3 are explained in Figure 2.

No specific permissions were required for the data from 18 locations/activities, and field studies did not involve dangerous activities. The 18 studied evacuation sites included Fukushima City (Fukushima Prefectural office, Azuma park, Kenpoku-Hoken-Fukushi office, Iino-gakushu Centre), Motomiya Town (Adatara-SA), Koriyama City (Big Palette, Koriyama General Gymnasium), Iwaki City (Iwaki-shi hokenjo, Nakoso-high school, Oura-elementary school, Umegaoka-meeting house), Kawamata Town (Kawamata-high school, Kawamata public office, Kawamata Gymnasium), Minami-soma City (Baji kouen, Satellite-Kashima), Iitate Village (Iitate public office), Kawauchi Village (Kawauchi-sonmin-taiiku centre) Marumori Town, Miyagi Prefecture (Machizukuri-centre, Hippo-junior high school), and were selected by the Ministry of Education, Culture, Sports, Science, & Technology in Japan (Figure 1). Surface contamination of evacuees was surveyed [4] and absorbed dose rates in the indoor and outdoor air were measured.

### Measuring instruments

Indoor and outdoor absorbed dose rates were measured at each evacuation site at a height of 1 m above the floor or ground surface using a 1×1-inch NaI(Tl) scintillation survey meter (TCS-171, Hitachi Aloka Medical, Co. Ltd., Japan). The measurement range of this device was 0.01–30 µGy/h, and measurement uncertainty was within ±15%. Measurements were repeated 3 times at each location with a 30-s time constant. Although the survey meter TCS-171 allows constants of 3, 10, or 30 s, the 30 s constant was chosen, which can be measured stably with the smallest counting error. The survey meter was calibrated in comparison with a 3×3-inch NaI(Tl) scintillation spectrometer (JSM-112, Hitachi Aloka Medical, Co. Ltd.) [1]. Gamma-ray pulse height distributions were obtained using a JSM-112 at each evacuation site. These measurements were performed 1 m above the floor or ground surfaces of indoor and outdoor areas, respectively. Measurement times were set at 900 s. Subsequently, gamma-ray pulse height distributions were unfolded using a 22×22 response matrix to evaluate absorbed dose rates in the air [5]. Calculations were performed on the assumption that fallout forms an infinite plane source on the ground.

### Statistical analysis

Statistical analyses were performed using the Origin software package (OriginLab Pro version 9.0, Northampton, MA, USA) and SPSS version 17.0 (IBM, Chicago, IL, USA) for Windows. Data were compared using Tukey–Kramer's test, and differences were considered significant when P<0.05.

## Results

### Dose reduction efficiency of structures

Dose reduction efficiency is defined as the ratio of the absorbed dose rate in the indoor air to that in the outdoor air. Public buildings that were used as evacuation sites included schools and exhibition halls, which were constructed of wood, aluminum, or reinforced concrete, but predominantly reinforced concrete (Figure 1). Between March 2011 and July 2011, dose reduction efficiencies of wood, aluminum, and reinforced concrete buildings were 0.55±0.04, 0.15±0.02, and 0.19±0.03, respectively (Table 1), 1.4 times higher than that reported by the International Atomic Energy Agency (IAEA) [6]. The reduction efficiency of reinforced concrete was similar to that in previous studies although that of aluminum structures has not been previously reported.

**Table 1:** Dose reduction efficiencies of gamma radiation in cloud or deposited radioactivity from the Fukushima Prefecture.

Structure	Dose reduction efficiency	
	Measurement data (March–July, 2011)	Reference [6]
Wooden construction	0.55±0.04^a^	0.4
Aluminum building construction	0.15±0.02	No data
Reinforced concrete construction	0.19±0.03	<0.2
Outdoor	1.0	1.0

Means ± S.E. of dose reduction efficiencies of wooden, aluminum, and reinforced concrete buildings were calculated 4 times on different days at 1 site, 15 times on different days at 3 sites, and 56 times on different days at 14 sites during March–July 2011.

a *P*<0.05 vs aluminum building and reinforced concrete construction by Tukey–Kramer's test.

**Figure 2:**
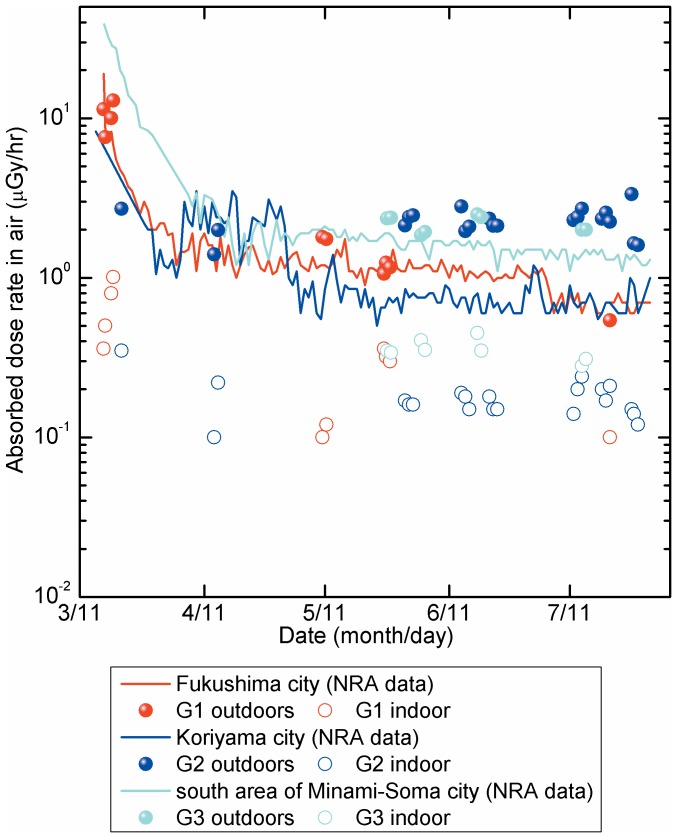
Variations in absorbed dose rates in the air immediately after the accident at F1-NPS. Dose rates in air were analyzed and calculated until July 2011 at G1, G2, and G3 sites (Figure 1). G1–G3 sites were measured 11, 21, and 10 times, respectively, on different days. The Nuclear Regulation Authority (NRA) data for each city are shown with lines (red: Fukushima city, blue: Koriyama city, light blue: south area of Minami-soma city), and values measured by our team are shown with circles. Solid and open circles represent outdoors and indoors, respectively.

**Figure 3:**
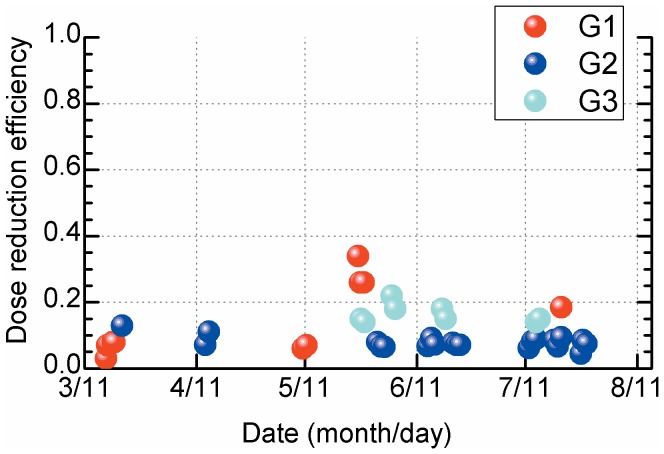
Dose reduction efficiency of G1–G3 sites (Figure 1). Data were calculated between March 2011 and July 2011. G1, G2, and G3 sites were measured 11, 21, and 10 times, respectively, on different days.

**Figure 4:**
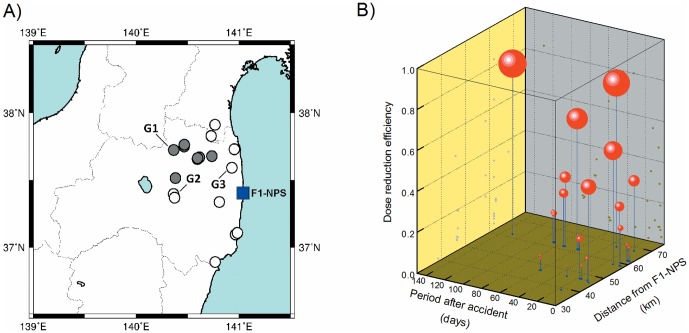
Dose reduction efficiencies at evacuation sites northwest of F1-NPS. A 3 dimensional representation of time variations, distances from F1-NPS, and dose reduction efficiencies at 8 evacuation sites northwest (gray) of F1-NPS is shown [A]. Sites with reinforced concrete construction were measured 27 times after the accident [B]. Sizes of spheres (red) reflect dose reduction efficiencies.

A high level of soil contamination with radioactive nuclides/compounds has been reported in the area northwest of F1-NPS [1], [3]. To assess the relationship between time variations of high absorbed dose rates in the outdoor air and dose reduction efficiency, 3 sites (G1, northwest; G2, west; G3, north of F1-NPS) were analyzed (Figure 1). According to measurements provided by the Nuclear Regulation Authority (NRA) in Japan (a third party organization that studies nuclear fuel facilities, research reactors, and nuclear waste storage and disposal facilities), absorbed dose rates in the outdoor air at each evacuation site decreased rapidly [7]. Absorbed dose rates in the outdoor air were 19, 8.3, and 39 µGy/h on March 16, 2011 and were 1.9, 2.8, and 1.8 µGy/h 4 weeks later. A further slight decrease was observed from April 2011 to July 2011 (Figure 2), and this was consistent with previously reported data. Despite decreased absorbed dose rates in the outdoor air, the time variation in dose reduction efficiency of various structure types was less than 0.2 at each site (Figure 3). However, dose reduction efficiency did appear to increase in proportion with distance from F1-NPS at 8 evacuation sites in the northwest (Figure 4).

## Discussion

We evaluated relationships between the time variations of absorbed dose rates in the indoor and outdoor air, distance from F1-NPS, and dose reduction efficiencies at 18 temporary evacuation sites in the Fukushima Prefecture. Dose reduction efficiencies of wooden structures were higher than those previously reported (IAEA-TECDOC-225, IAEA-TECDOC-1162), whereas those of reinforced concrete were similar [6], [8]. Yoshida reported that Japanese wooden constructions have a dose reduction efficiency of 0.36–1.0, assuming that absorbed dose rates in the indoor air depend on the shielding effect of each Japanese dwelling and on indoor surface contamination [9]. To accurately calculate individual exposures, further information about the surrounding factors at wooden buildings is required.

The dose reduction efficiency of aluminum structures was similar to that of reinforced concrete (Table 1, Figure 2), and aluminum and concrete perform similarly in terms of corrosion resistance and incombustibility. Aluminum construction is lightweight and is also cheaper than concrete because of the absence of steel rods [10]. Hence, aluminum structures may be employed as gamma-radiation shelters in the future.

No large variations in dose reduction efficiencies were observed at sites G1–G3, whereas absorbed dose rates in the air decreased drastically during the 5 months after the accident (Figure 3). There is no background data available before the accident for the sites G1–G3; however, in 1981, Abe *et al*. reported background levels of 75, 81, and 84 nGy/h at Fukushima (near site G1), Koriyama (near site G2), and Minami-soma cities (near site G3), respectively [11]. Thus, these values were subtracted from the raw NRA data and calculated half-lives (HFs) as the “environmental half-life” of artificial radioactive nuclides (Table 2) using the least squares method. Environmental HFs of the early term (early stage) were 1.6±0.1, 5.7±1.3, and 4.1±0.1 days at sites G1, G2, and G3, respectively. HF at G1 was significantly shorter than that at G3, which was shorter than that at G2 (Tukey–Kramer's test). Environmental HFs of the delay term at 4 months after the accident (delay stage) were significantly longer at G3 and were similar at G1 and G2 (G1, 71±8.1 days; G2, 68±19 days; G3, 170±37 days; Tukey–Kramer's test). These variations may reflect a mixture of artificial radioactive nuclides with long and short physical HFs, and that the deposition of iodine-131, iodine-132, tellurium-132, caesium-134, caesium-136, and caesium-137 onto soils is dependent on physicochemical forms and size distributions [1], [4], [12], [13]. In the Chernobyl accident, the environmental HF of ^137^Cs-compounds was shown to be 3–4 years on lichen species [14], [15]. However, environmental HFs of these compounds at sites G1–G3 were shorter than those in these reports because surface contamination on asphalt is easily washed away. Thus, the present absorbed dose rates at 5 months after the accident do not contradict those reported at that time by the government and in previous scientific papers.

**Table 2:** Environmental half-lives of outdoor radioactive molecules at each evacuation site.

Location	Direction	Distance	Early stage	Delay stage
	from F1-NPS	[km]	[days]	[days]
G1	Northwest	62	1.6±0.1^a^	71±8.1^c^
G2	West	60	5.7±1.3^b^	68±19*^d^*
G3	North	21	4.1±0.1	170±37

Environmental half-lives (HFs) at cities of evacuation sites G1–G3 (Figure 1) were calculated using the least squares method with the obtained data;

a *p*<0.01 vs. early stage (15–31 March) of HFs at G2 and G3;

b *p*<0.05 vs. early stage of HFs at G3;

c *p*<0.05 vs. delay stage (April–July) of HFs at G3, and *^d^p*<0.05 vs. delay stage of HFs at G3; by Tukey–Kramer's test.

At the evacuation sites in the northwest, dose reduction efficiency was not maintained and did not reflect time variations and distances from F1-NPS (Figure 4). Thus, we assumed that indoor dose reduction efficiency is reduced by surface contamination following repeated entry and exit of evacuees [9]. Moreover, indoor contamination may explain this decrease because high absorbed dose rates in the air have been observed in the northwest areas such as Namie Town (24 km from F1-NPS) and Iitate-Mura (30–40 km from F1-NPS) where the dose rates were 32 and 16 µGy/h, respectively, on July 31, 2011 and were 0.03 µGy/h in the area northwest of F1-NPS before the accident [4]. Previous reports have estimated effective doses and local exposure doses on the basis of behavior data [16]–[18]. These data can be used to identify measures that reduce individual exposure and to calculate individual doses following future accidents. However, numerous additional factors must be considered and continuous measurements must be made to accurately calculate individual exposure doses.

In conclusion, the relationship between dose reduction efficiencies of evacuation sites, time, and distances of evacuation sites from the accident depend on building structure types, levels of contamination, and evacuee behaviors. These factors warrant further studies of radiation safety management.
